# Insulin-like Growth Factor Binding Proteins and Cellular Senescence Are Involved in the Progression of Non-Alcoholic Fatty Liver Disease and Fibrosis in a Mouse Model

**DOI:** 10.3390/medicina60030429

**Published:** 2024-03-02

**Authors:** Carolina Guzmán, Miriam G. Bautista-Ubaldo, Adriana Campos-Espinosa, Ivette I. Romero-Bello, Ángel Daniel Santana-Vargas, Gabriela Gutierrez-Reyes

**Affiliations:** 1 Laboratorio de Hígado, Páncreas y Motilidad, Unidad de Medicina Experimental, Facultad de Medicina, Universidad Nacional Autónoma de México, Hospital General de México “Dr. Eduardo Liceaga”, Mexico City 06720, Mexico; mirigisel.2610@gmail.com (M.G.B.-U.); adria.camp88@hotmail.com (A.C.-E.); gabgurey@yahoo.com.mx (G.G.-R.); 2 Unidad de Medicina Experimental, Dirección de Investigación, Hospital General de México “Dr. Eduardo Liceaga”, Mexico City 06720, Mexico; danievsan@gmail.com

**Keywords:** insulin-like growth factor binding proteins, liver fibrosis, cellular senescence, steatohepatitis, steatosis

## Abstract

*Background and Objectives*: Non-alcoholic fatty liver disease (NAFLD) is highly prevalent worldwide. It progresses from simple steatosis to non-alcoholic steatohepatitis (NASH). Fibrosis is often present during NAFLD progression; however, factors determining which subjects develop NASH or fibrosis are unclear. Insulin-like growth factor binding proteins (IGFBPs) are a family of secreted proteins involved in senescence and scarring, mainly synthetized in the liver. Here, we aimed to study the association of IGFBPs and their induced senescence with the progression of NAFLD and liver fibrosis. *Materials and Methods*: A total of 16-week-old male C57BL/6 mice weighing 23 ± 3 g were fed either methionine/choline-deficient (MCD) or control diet for 2, 8, or 12 weeks. Blood and liver samples were collected, and a histological assessment of NAFLD and fibrosis was performed. Fat contents were measured. Cellular senescence was evaluated in the liver. IGFBP levels were assessed in the liver and serum. Data were expressed as mean ± SD and analyzed by a one-way ANOVA followed by Tukey’s test. Lineal regression models were applied for NAFLD and fibrosis progression. *p* < 0.05 was considered significant. *Results*: IGFBP-1 and -2 were increased in serum during NAFLD. IGFBP-7 was significantly increased in the serum in NASH compared with the controls. Senescence increased in NAFLD. Serum and liver IGFBP-7 as well as SA-β-gal activity increased as fibrosis progressed. Both IGFBP-7 and cellular senescence were significantly higher during NAFLD and fibrosis in MCD-fed mice. *Conclusions*: IGFBP-1, -2, and -7, through their consequent senescence, have a role in the progression of NAFLD and its associated fibrosis, being a plausible determinant in the progression from steatosis to NASH.

## 1. Introduction

Non-alcoholic fatty liver disease (NAFLD) is the most prevalent hepatic disease worldwide, and it is highly associated with metabolic disorders, including obesity, insulin resistance, diabetes, and metabolic syndrome [1,2,3]. NAFLD comprises a spectrum of histopathological changes characterized by an abnormally high accumulation of lipids in the liver found in simple steatosis (SS) that progresses to steatohepatitis (NASH), where inflammation and ballooning are observed [1]. Fibrosis might be present or absent during both simple steatosis and NASH [4]; however, factors determining which patients develop fibrosis are not clear, although metabolic, endocrine, genetic, and aging have been suggested among them [1]. Cirrhosis, hepatocellular carcinoma, and liver failure are the hepatic endpoints of this disease, but the presence of severe fibrosis is considered the most important predictor of NAFLD outcomes and death risk [5,6,7].

Insulin-like growth factor (IGF) binding proteins (IGFBPs) are a family of secreted proteins whose primary function is binding IGFs in the bloodstream, regulating their bioavailability and half-life [8,9]. IGF-independent functions, including proliferation, apoptosis, and cellular senescence, have been described [8,9]. These proteins are expressed in most tissues, but the liver is the main source for most of them [9]. A role in chronic liver disease has been suggested for some IGFBPs. IGFBP-1 expression is increased in the liver during post-injury regeneration [10] and is considered a hepatoprotective factor that prevents apoptosis in hepatocytes [11]. In humans, IGFBP-3 expression is reduced in hepatocellular carcinoma compared with cirrhotic tissue. IGFBP-3 is regulated by transforming growth factor (TGF)-β, promoting hepatic stellate cells (HSCs) migration in vitro and increasing portal hypertension in the bile duct ligation model [12], and it might be a key protein during alcoholic liver disease by inducing lipid droplet and triglyceride accumulation in vitro [13]. IGFBP-7, a low IGF-affinity protein, is mainly expressed in HSC, being upregulated during transdifferentiation [14] and activation [15,16]. The expression of IGFBP-7 is significantly elevated in liver biopsies from patients with fibrosis and cirrhosis [17], whereas the inhibition or silencing of this protein prevents the accumulation of an extracellular matrix in experimental models of liver fibrosis [16,17]. IGFBP-7 has been shown to induce cellular senescence [18]; in fact, IGFBP-7 expression in HCC tissue is lower compared with healthy tissues [19]. Regarding metabolic abnormalities, including NAFLD, low levels of IGFBP-2 have been reported in obesity, type 2 diabetes, and metabolic syndrome [20], whereas IGFBP-5 is increased in NASH [21]. Serum IGFBP-3 is decreased in patients with NAFLD [22], whereas the IGF-1/IGFBP-3 ratio has been associated with a lower likelihood of NAFLD, lower-grade steatosis [23], and histopathologic features of the liver biopsy, including ballooning and inflammation [24]. Interestingly, an increased expression of IGFBP-1 and IGFBP-7 in the liver might contribute to hepatic insulin resistance [25] and further fat accumulation.

Accordingly, IGFBPs might have a role during NAFLD as well as in the development of fibrosis; however, this is not clear. The aim of this study was to assess IGFBPs in both the serum and liver of mice during the progression of NAFLD and the onset of fibrosis in this disease, and we hypothesized that IGFBPs, through their IGF-independent actions, might have an actual role in the development of the disease.

## 2. Materials and Methods

### 2.1. NAFLD Induction

Male C57BL/6 mice of 16 weeks of age and weighing 23 ± 3 g were obtained from the Animal Care Facilities at the Experimental Medicine Unit from the School of Medicine, UNAM at Hospital General de México, and maintained under controlled conditions. Both food and water were allowed ad libitum. All animals received humane care; all procedures were approved by the Institutional Committee of Care and Use of Laboratory Animals (FM/DI/005/2022 approved on 6 September 2022) and agreed with the national guidelines and the ARRIVE guidelines for animal use and care in research. In order to develop different stages of NAFLD, mice were randomly assigned to be fed either a methionine/choline-deficient (MCD) or a methionine/choline complete (Control) diet (MP Biomedicals, CA, USA) for 2 (MCD 2w, *n* = 19; control 2w, *n* = 7), 8 (MCD 8w, *n* = 17; control 8w, *n* = 9), or 12 weeks (MCD 12w, *n* = 20; control 12w, *n* = 8).

### 2.2. Sample Collection

After the appropriate time, mice were anesthetized with xylazine/ketamine. Blood was collected by cardiac punction and allowed to clot at 4 °C; serum was obtained by centrifugation at 750× *g* for 10 min and stored at −80 °C until it was assayed. Liver samples from the left and medial lobes were collected, snap frozen in liquid nitrogen, and stored at −80 °C until they were assayed. Samples from the left lobe were divided to either be fixed in 3.7% formaldehyde/phosphate-buffered saline (PBS) and embedded in paraffin or, to be embedded in Tissue-tek OCT (Sakura Finetek, CA, USA) and stored at −20 °C until assayed. 

### 2.3. Histological Assessment

A histological evaluation of fatty liver disease was performed in hematoxylin/eosin-stained sections according to the non-alcoholic fatty liver disease activity score (NAS) [26]. Fibrosis was assessed in Masson’s Trichrome-stained sections and classified according to their fibrosis degree [26]. Liver fat contents were assessed in frozen sections stained with Oil-Red O (Abcam, Waltham, MA, USA) and quantified by a morphometric analysis using Image J v1.53k software (NIH, Bethesda, MD, USA), according to [27]. Briefly, 10 optic fields were captured, and the percentage of the red-stained area was calculated as a percentage of the complete area of the optic field.

### 2.4. Senescence Analysis

Cellular senescence was assessed in the frozen sections embedded in Tissue-Tek OCT using the Senescence Detection Kit (Abcam, Waltham, MA, USA). A blue stain was indicative of the activity of the senescence-associated β-galactosidase (SA-β-gal). A morphometric analysis was performed using Image J v1.53k software.

### 2.5. Liver Protein Isolation

Total protein was obtained from each sample using the PBS-Protease Inhibitor Cocktail Set III (Calbiochem, Darmstadt, Germany). Protein integrity was tested by SDS-PAGE prior to the assays.

### 2.6. IGFBPs Assessment

IGFBP-1, -2, -3, -5, -6, and -7 from the livers and serum were quantified by multiple suspension arrays (Milliplex MAP, MIGFBPMAG43K, Merck-Millipore, Billerica, MA, USA), according to manufacturer instructions. Bead regions were assigned as follows: IGFBP-1: 27; IGFBP-2: 39; IGFBP-3: 42; IGFBP-5: 55; IGFBP-6: 61; and IGFBP-7: 72.

### 2.7. Statistics

Data were analyzed by SSPS v22 and presented as mean ± standard deviation (SD). Subjects receiving the control diet did not exhibit differences attributable to the time of exposure to the diet or aging and were analyzed as one single control group. Subjects receiving the MCD diet were categorized according to NAS or fibrosis stages. A one-way ANOVA followed by Tukey’s post-hoc test was performed. Independent linear regression models for the stage of NAFLD and fibrosis were used to explore the associations between the time of exposure to the MCD diet, the degree of fibrosis, and the levels of IGFBPs (both crude and adjusted by hepatic lipid content). *p* < 0.05 was considered significant.

## 3. Results

### 3.1. Histological Classification

**Figure 1:**
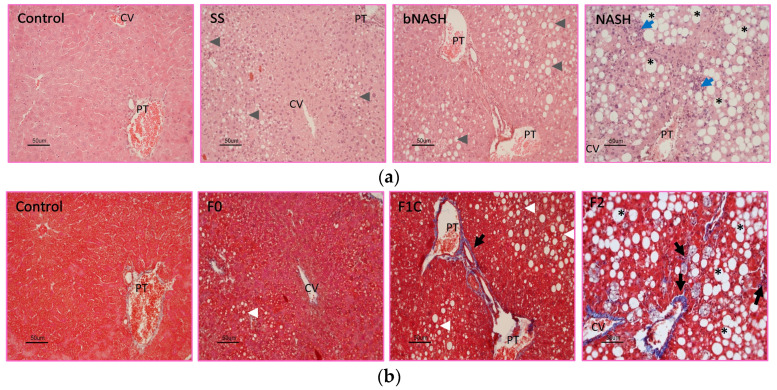
NAFLD stages and fibrosis. Mice were fed either methionine/choline-deficient or control diet for 2, 8, or 12 weeks. Liver sections were stained with hematoxylin/eosin or Masson’s trichrome to assess NAS and fibrosis degree, respectively. (**a**) Progression of NAFLD (SS: simple steatosis; bNASH: borderline NASH; NASH: definite NASH); (**b**) fibrosis progression (F0: no fibrosis; F1C: portal fibrosis; F2: portal and perisinusoidal fibrosis). Bar = 50 µm. CV: central vein; PT: portal triad; * ballooning; blue arrow: inflammation; black arrow: fibrosis; arrowhead: steatosis.

Subjects from the different times of treatment were classified by NAS. Accordingly, all 24 subjects fed the control were classified as healthy (control; NAS = 0). From MCD-fed mice, 10 were classified as simple steatosis (SS, NAS = 1–2), 27 as borderline NASH (bNASH; NAS = 3–4), and 19 as definite NASH (NASH; NAS = 5–8). The representative micrographs are shown in Figure 1a. None of the control subjects exhibited fibrosis. In contrast, from MCD-fed subjects, 7 were F0 (no fibrosis), 27 were F1C (portal fibrosis), and 22 were F2 (portal and perisinusoidal fibrosis); no subjects with F3 or cirrhosis were found in this study. Figure 1b shows representative micrographs of the stages of fibrosis.

### 3.2. Liver Fat Contents

**Figure 2:**
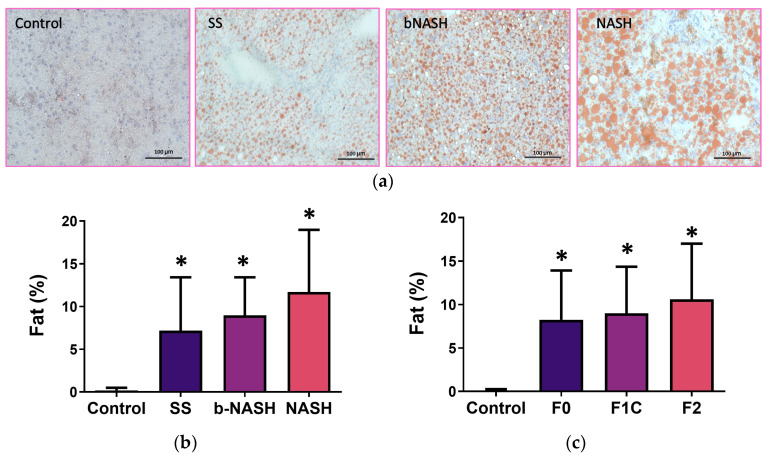
Hepatic lipid contents. Mice were fed either methionine/choline-deficient or control diet for 2, 8, or 12 weeks. Livers were collected, and frozen sections were stained with Oil-Red O. A morphometric assessment of lipid contents was performed and associated with the stage of NAFLD, or the fibrosis degree. (**a**) Micrographs showing the lipid accumulation (neutral lipids appear stained in red) observed during NAFLD progression. (**b**) Percentage of lipids according to the NAFLD stage. (**c**) Percentage of lipids according to the fibrosis degree. SS: simple steatosis; bNASH: borderline non-alcoholic steatohepatitis; NASH: non-alcoholic steatohepatitis. Mean ± SD. One-way ANOVA followed by Tukey’s post-hoc test. * *p* < 0.05. Bar = 100 µm.

Fat contents were significantly higher in MCD-fed mice compared with the controls, regardless of the time of consumption, the stage of NAFLD, or fibrosis degree (Figure 2a). No differences in fat were observed among SS, bNASH, and NASH (Figure 2b). Consistently, when analyzed according to the fibrosis degree, increased fat was observed in MCD-fed mice; however, no differences in the fat contents were found from F0 to F2 (Figure 2c).

### 3.3. IGFBP Levels in NAFLD

#### 3.3.1. Liver Tissue

**Figure 3:**
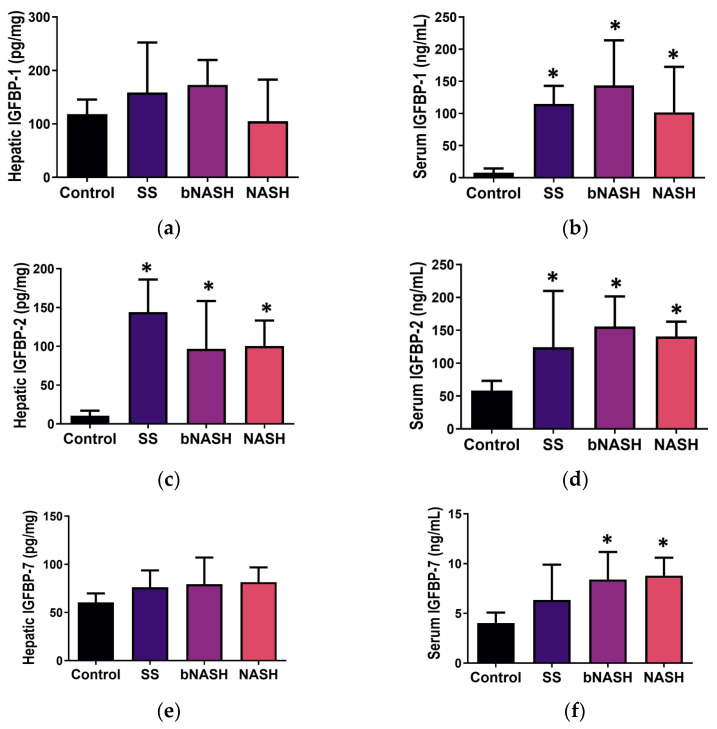
IGFBP levels during NAFLD. Mice were fed either a methionine/choline-deficient (MCD) or control diet for 2, 8, or 12 weeks. Hepatic and serum levels of IGFBPs were assessed and analyzed according to the stage of NAFLD evaluated by NAS. (**a**) Hepatic expression of IGFBP-1; (**b**) IGFBP-1 serum levels; (**c**) IGFBP-2 hepatic expression; (**d**) IGFBP-2 serum levels; (**e**) IGFBP-7 hepatic expression; (**f**) IGFBP-7 serum levels. Mean ± SD. One-way ANOVA followed by Tukey’s post-hoc test. * *p* < 0.05. SS: simple steatosis; bNASH: borderline NASH; NASH: definite NASH.

IGFBPs were analyzed in the liver and serum according to the stage of NAFLD. In the liver, IGFBP-2 expression was significantly increased during all NAFLD stages compared to the controls (Figure 3c), whereas IGFBP-3 expression was higher in bNASH compared with SS and NASH (control = 88.6 ± 12.1, SS = 67.0 ± 14.2, bNASH = 110.5 ± 30.3, NASH = 66.0 ± 18.5 pg/mg of liver; *p* < 0.05), and other IGFBPs did not exhibit any change in the hepatic tissue related to NAFLD progression. However, using linear regression models, statistically significant associations were observed. In both crude and adjusted hepatic lipid contents, IGFBPs were predictor of NAFLD stage as follows: IGFBP-1 for bNASH and IGFBP-2 for every NAFLD stages (SS, bNASH, and NASH) (Table 1). A crude significant predictor in the liver was IGFBP-6 (SS and NASH), whereas IGFBP-3 adjusted by lipids was a predictor for NASH (Supplementary Table S1).

#### 3.3.2. Serum

In the serum, IGFBP-1 and -2 were increased in all NAFLD stages, but no differences were observed among them (Figure 3b,d); IGFBP-7 was increased during both borderline and definite NASH compared with the controls (Figure 3f). IGFBP-3, -5, and -6 serum levels did not exhibit differences related to NAFLD. When analyzed by linear regression (Table 1) and both crude and adjusted lipid contents, serum IGFBP-1 was an independent predictor of SS, bNASH, and NASH, whereas IGFBP-2 and -7 were predictors for bNASH. Crude significant predictors in serum were IGFBP-2 (SS and NASH) and IGFBP-7 (SS). When adjusted by lipids, predictors in serum were IGFBP-3 (bNASH and NASH; Supplementary Table S1) and IGFBP-7 (NASH). Table 1 shows the coefficients (95% CI) for the associations that were significant for the stages of NAFLD in the crude analysis and remain significant after adjusting by hepatic lipid contents; these associations were confirmed for IGFBP-1, -2, and -7. Interestingly, analyzing according to the time of exposure to the MCD diet, hepatic IGFBP-2 (crude and adjusted), and serum levels of IGFBP-2 and -7 (crude) were shown to be predictors for the progression of NAFLD.

### 3.4. IGFBP during Mild-to-Moderate Fibrosis in NAFLD

#### 3.4.1. Liver Tissue

**Figure 4:**
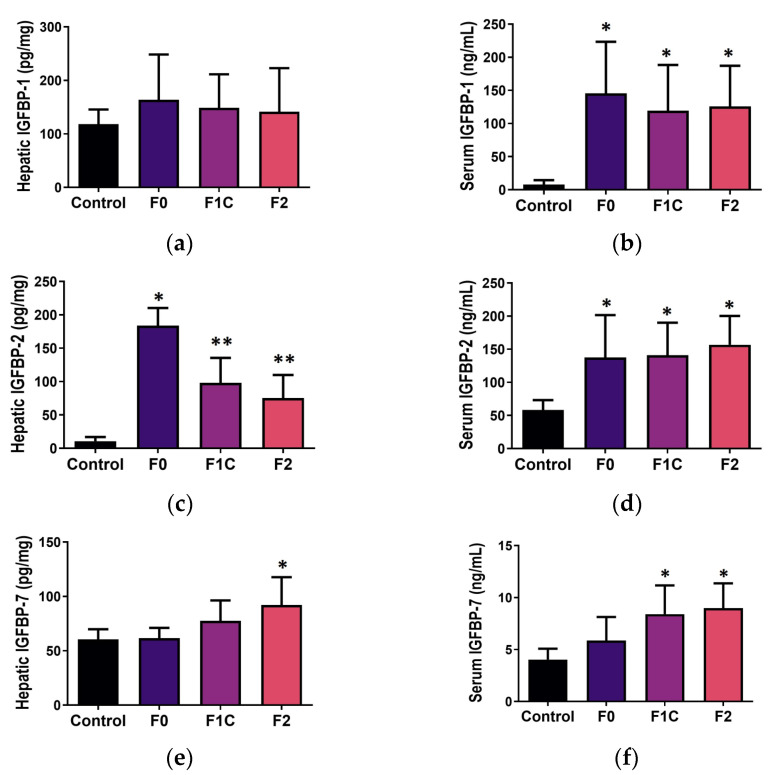
IGFBP during liver fibrosis in NAFLD. Mice were fed either a methionine/choline-deficient (MCD) or control diet for 2, 8, or 12 weeks. Hepatic and serum levels of IGFBPs were assessed and analyzed according to the fibrosis degree during NAFLD. (**a**) IGFBP-1 hepatic expression; (**b**) IGFBP-1 serum levels; (**c**) IGFBP-2 hepatic expression; (**d**) IGFBP-2 serum levels; (**e**) IGFBP-7 hepatic expression; (**f**) IGFBP-7 serum levels. Mean ± SD. One-way ANOVA followed by a Tukey post-hoc test. * *p* < 0.05 vs. control; ** *p* < 0.05 vs. control and F0. SS: simple steatosis; bNASH: borderline NASH; NASH: definite NASH.

Some IGFBPs have been related to fibrosis in different tissues [28,29,30,31]; here, in MCD-induced NAFLD, we observed an increase in the expression of IGFBP-2 and IGFBP-7 in the liver (Figure 4c,e). IGFBP-2 was increased in all MCD-fed mice compared with the controls; according to fibrosis, F0 exhibited the highest expression in the hepatic tissue, and it decreased in F1C and F2 (Figure 4c). Regarding IGFBP-7, this protein was increased in F2 compared with the controls and F0, but no differences were observed when compared with F1C (Figure 4e). Similarly, according to the degree of fibrosis, statistically significant associations were observed in the liver, for both crude and adjusted by hepatic lipids, in IGFBP-2 (F0, F1C, and F2) and IGFBP-7 (F2), whereas a crude predictor was IGFBP-5 (F0 and F1C; Supplementary Table S2), and IGFBP-1-adjusted by lipids was a significant predictor for F0 and F2 (Table 2).

#### 3.4.2. Serum

In the serum, IGFBP-1 and -2 were increased in all MCD-fed mice, but no differences were observed among the fibrosis degrees (Figure 4b,d); IGFBP-7 was increased in mice with fibrosis, F1C, and F2, compared with controls (Figure 4f). IGFBP-3, -5, and -6 serum levels did not exhibit differences related to fibrosis. Linear regression models showed a statistically significant association in both crude and adjusted by lipids for IGFBP-1 (F0, F1C, and F2), IGFBP-2 (F1C and F2), and IGFBP-7 (F1C and F2); IGFBP-2 was a crude predictor for F1C (Table 2) and IGFBP-6 for F2 (Supplementary Table S2). Regarding the time of exposure to the MCD diet, a significant association with fibrosis was observed only for serum levels of IGFBP-2 (crude). Table 2 shows the coefficients (95% CI) for the associations that were significant for the fibrosis degree in the crude analysis and remain significant after adjusting for lipids.

### 3.5. Cellular Senescence during NAFLD and Fibrosis

**Figure 5:**
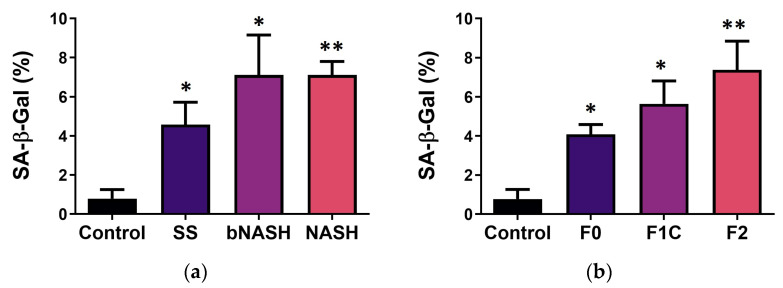
Cellular senescence in NAFLD and fibrosis. Non-alcoholic fatty liver disease was induced by the MCD diet. Cellular senescence was evaluated in liver frozen sections as the percentage of activity of SA-β-Gal. (**a**) Cellular senescence during NAFLD. (**b**) Cellular senescence during liver fibrosis associated with NAFLD. Mean ± SD. One-way ANOVA followed by a Tukey post-hoc test. * *p* < 0.05 vs. control; ** *p* < 0.05 vs. control and F0. SS: simple steatosis; bNASH: borderline NASH; NASH: definite NASH.

The observation of IGFBP-7 increased in serum in both borderline and definite NASH stages as well as during the F1C and F2 fibrosis stages, combined with its identification as a predictor by both crude and adjusted lipid models, suggests a possible role for this protein in the progression of NAFLD. We assessed cellular senescence as a mechanism mediated by IGFBP-7, which has been shown to be involved in the progression of chronic liver disease [32,33]. As expected, cellular senescence was increased during NAFLD compared with the healthy controls. SA-β-gal activity was significantly increased in SS subjects compared with the controls, and it was significantly higher in bNASH and NASH compared with the controls and SS (Figure 5a). Regarding fibrosis, cellular senescence progressively increased during fibrosis compared with the healthy controls. When analyzed according to the fibrosis stages, F2 exhibited a higher percentage of senescent cells compared with F0 and F1C (Figure 5b), suggesting that IGFBP-7 and its induced senescence might have a role in the progression from mild-to-moderate fibrosis during NAFLD. 

## 4. Discussion

The IGFBP superfamily has been related to insulin resistance [25,34], fat deposition, and other cellular processes, including senescence [9]. All these factors have a clear role in the development of NAFLD. Here, we assessed a group of IGFBPs in both the liver and serum according to the progression of NAFLD and fibrosis in the MCD mouse model. Our results show significantly increased serum levels of IGFBP-1 and -2 in mice with NAFLD and fibrosis. IGFBP-7, a low-affinity IGFBP, was increased in the serum as well as in the liver tissue; interestingly, it was elevated in the serum in bNASH and NASH as well as during fibrosis in F1C and F2, suggesting a role for IGFBP-7 in the progression of NAFLD and the onset of fibrosis in this model. Linear regression models confirmed the predictive value of IGFBP-1, -2, and -7 for both NAFLD stages and fibrosis degree.

As expected, we observed higher fat contents in the liver of all NAFLD subjects compared with the controls, although no differences in the amounts of fat were observed among the stages of the disease (Figure 2b) or among the different degrees of fibrosis observed: F0 to F2 (Figure 2c).

For IGFBP-1, we observed differences in the serum related to both NAFLD and fibrosis (Figure 3b and Figure 4b). This protein is known to possess a hepatoprotective role [11]; IGFBP-1 is among the first genes to be overexpressed after a partial hepatectomy [10], and it also increases during liver disease [35,36]. IGFBP-1 is transcriptionally regulated by insulin [37], and it has been linked, as well as IGFBP-2, to insulin sensitivity [38]. On the other hand, it has been shown that hedgehog, a well-known fibrogenic pathway, also regulates IGFBP-1 expression [39], suggesting that this peptide might also be a fibrogenic mediator in the liver. Serum levels of IGFBP-1 were a significant crude and adjusted predictor for SS, bNASH, and NASH (Table 1). Regarding fibrosis, hepatic IGFBP-1 was an adjusted predictor for F0 and F2, whereas the serum levels of this protein were significant predictors for F0, F1C, and F2. We consider these associations to be related to both the hepatic lipid contents and the onset of liver fibrosis, and according to Hagstrom et al., IGFBP-1 levels might increase even more as fibrosis progresses [29].

IGFBP-2 has been extensively studied in obesity, type 2 diabetes, insulin resistance, metabolic syndrome [34,40,41,42,43], and as an inductor of cancer [44]. In fact, this peptide has been suggested as a possible protector against obesity and insulin resistance [45]. However, not much data are available concerning NAFLD. Here, we observed an increase in IGFBP-2 during NAFLD and fibrosis in both the liver and serum. Interestingly, during fibrosis, IGFBP-2 was significantly elevated in the liver during F0 compared with F1C and F2; however, both stages exhibited higher expression compared with the controls. The hepatic expression of IGFBP-2 was a significant crude and adjusted predictor of SS, bNASH, and NASH, whereas its serum levels were crude predictors as well. The increased levels of IGFBP-2 observed in the MCD-induced NAFLD might be explained by the fact that this model is characterized by a low bodyweight and a lack of insulin and leptin resistance. However, the effect observed in the liver during fibrosis, where this peptide was a significant predictor of fibrosis progression in both crude and adjusted lipid models, is noteworthy. Although IGFBP-2 has been suggested to have a role in idiopathic pulmonary fibrosis [30,31], its role in hepatic fibrosis is not clear.

Regarding IGFBP-7, this peptide is a well-known tumor suppressor. This peptide is downregulated in hepatocellular carcinoma lesions, compared with its expression in adjacent healthy tissue [19], and in IGFBP-7-deficient mice, the spontaneous development of liver tumors is observed [46]. IGFBP-7’s ability to induce cellular senescence [46,47,48] has been suggested as the anti-tumoral mechanism, and it is implicated in HSC activation [17,49,50], which in turn produces ECM accumulation and fibrosis [15,16]. In mice, the deletion of IGFBP-7 attenuates liver fibrosis [51]. Hepatocyte cellular senescence has also been related to HSC activation and NAFLD as well [33,52]. In our study, IGFBP-7 was significantly increased in the serum of subjects with bNASH and NASH compared with the controls (Figure 3f). IGFBP-7 progressively increased in both the liver and serum according to fibrosis; in the liver, its expression was significantly higher in F2 (Figure 4e), whereas its serum levels rose in F1C and F2 (Figure 4f). When adjusted by the lipid content in the liver, serum IGFBP-7 was a predictor of NASH (Table 1). Regarding fibrosis, IGFBP-7 was a predictor (crude and adjusted by lipids) of F2 (Table 2).

Here, we show that IGFBP-7 levels were in accordance with cellular senescence, assessed as the percentage of activity of SA-β-gal. During NAFLD progression, higher percentages of senescent cells were detected in bNASH and NASH compared with the controls and SS. However, SS also exhibited increased levels of senescent cells compared with the controls (Figure 5a). In a similar manner, SA-β-gal activity was augmented as fibrosis progressed from mild to moderate, but high levels of senescent cells were also observed in F0 (Figure 5b). Fat depots have also been implicated in the induction of cellular senescence in the affected hepatocytes as a consequence of lipotoxicity, increased oxidative stress, DNA damage, and telomere erosion [33]. Our data agree with the association between increased fat contents in hepatocytes and exhibiting a higher percentage of senescence [33]. However, as seen in SS and F0, hepatic fat depots are not enough to induce the progression of NAFLD, nor fibrosis; instead, the increase in IGFBP-7 followed by cellular senescence might be a trigger to progress to NASH, as well as the onset of ECM accumulation, by activating HSC. Senescence is known to increase in aging organs and tissues [53]; however, we do not consider aging a factor affecting our results since we did not observe differences in the SA-β-gal activity in the livers of mice fed a control diet for 2, 8, or 12 weeks, even though they were 6–10 weeks older at sample collection. In fact, they were reported as a single control group. Cellular senescence is involved in a range of chronic liver disorders, including viral hepatitis B and C [54], alcoholic liver disease [32], genetic haemochromatosis [55], and NAFLD [33]; however, the mechanisms inducing senescence in chronic liver disease remain unclear. NAFLD and its derived fibrosis occur simultaneously; however, not all subjects progress at the same rate, and fibrosis is not strictly associated with NASH [4]. Serum levels of IGFBP-7 were significantly increased in the subjects with fibrosis (F1C and F2), but its hepatic expression was only increased in the F2 livers. Regarding senescence and fibrosis, we observed higher percentages of activity of SA-β-gal in the liver from all MCD-fed subjects that increased with the fibrosis progression.

**Figure 6:**
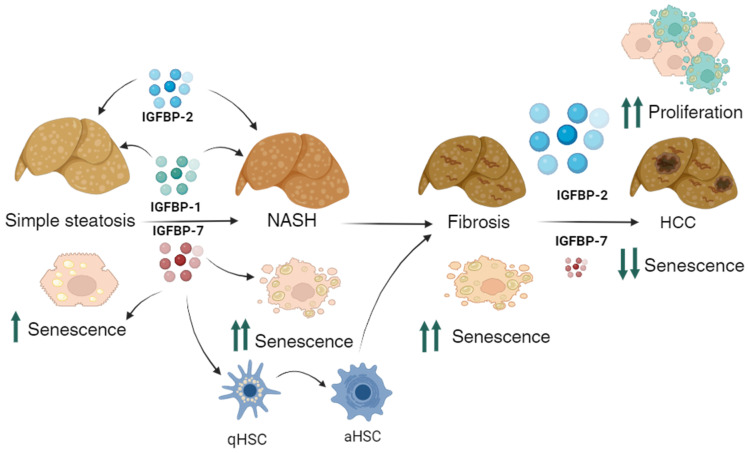
Suggested role of IGFBPs during the progression of NAFLD and fibrosis. In the early stages of NAFLD, lipid accumulation induces IGFBP expression. IGFBP-7 induces cellular senescence and progression to NASH, as well as hepatic stellate cell (HSC) activation and fibrosis. During severe fibrosis, this peptide is downregulated, probably by methylation; without IGFBP-7, senescence is insufficient to prevent proliferation, which is stimulated by IGFBP-2; thus, hepatocellular carcinoma (HCC) begins. Arrows pointing up: increase, arrows pointing down: decrease.

Several factors determine which subjects develop fibrosis during NAFLD: the well-documented oxidative stress [56], endoplasmic reticulum stress, and, as suggested by our findings, IGFBP-7 and its resulting cellular senescence. Other features of NAFLD, including inflammation and ballooning, might be implicated in the increased senescence observed in bNASH and NASH. In our results, increased IGFBP-1, -2, -7, and senescence were the most important determinants for the progression to NASH and fibrosis in the MCD-induced NAFLD mouse model, as shown in the suggested mechanism in Figure 6.

One limitation of our study is that we observed only mild-to-moderate fibrosis; further studies in severer stages of fibrosis, cirrhosis, and hepatocellular carcinoma derived from NAFLD are needed in order to establish a complete association between fibrosis, IGFBP-7, and senescence during NAFLD. Another limitation is in the MCD model, where NAFLD occurs in the absence of metabolic affection; however, we consider our data valuable since the presence of such metabolic affections might also influence IGFBPs expression as well as senescence, which is a confounding factor when relating them to hepatocellular lipid contents and fibrosis.

## 5. Conclusions

In conclusion, IGFBP-1, -2, and -7 increases were significant predictors for the progression of NAFLD and fibrosis in the MCD mouse model. Particularly IGFBP-7, a molecule involved in the activation of HSC and accumulation of ECM and a well-known inductor of cellular senescence, might be a determinant for the progression to NASH and fibrosis. Both IGFBP-7 and its consequent senescence have a role in the progression from SS to NASH and during the onset of fibrosis and its progression from mild to moderate.

## Figures and Tables

**Table 1:** Linear regression models according to the NAFLD stage.

		IGFBP-1		IGFBP-2		IGFBP-7	
		Crude	Adjusted	Crude	Adjusted	Crude	Adjusted
Liver tissue	Exposure	−3.195 (−26.99, 20.60)	13.519 (−11.41, 38.45)	−21.593 (−38.68, −4.50)	−27.348 (−47.76, −6.93) *	5.875 (−2.50, 14.25)	9.258 (−0.01, 18.52)
	SS	38.493 (−25.85, 102.84)	62.778 (−18.88, 144.43)	122.548 (78.25, 166.84) ***	116.826 (52.09, 181.56) **	18.722 (−3.93, 41.38)	24.296 (−6.05, 54.65)
	bNASH	55.529 (9.35, 101.71) *	79.380 (12.88, 145.88)	94.752 (61.80, 127.70) ***	107.422 (46.69, 168.16) **	17.358 (1.10, 33.62)	10.902 (−13.81, 35.62)
	NASH	3.672 (−51.09, 58.44)	28.816 (−51.37, 109.01)	89.745 (52.11, 127.38) ***	105.661 (38.16, 173.16) **	21.080 (1.80, 40.36)	10.560 (−19.24, 40.36)
Serum	Exposure	−17.108 (−38.35, 4.13)	−9.390 (−33.877, 15.10)	23.904 (8.63, 39.17) ***	14.966 (−2.18, 32.11)	−0.013 (−1.00, 0.97) *	0.277 (−0.94, 1.49)
	SS	100.763 (45.55, 155.98) **	138.635 (53.61, 223.66) **	97.291 (57.60, 136.98) ***	56.635 (−2.89, 116.16)	3.890 (1.32, 6.46) **	3.433 (−0.79, 7.65)
	bNASH	144.465 (101.25, 187.68) ***	187.488 (126.29, 248.69) ***	86.343 (55.28, 117.41) ***	52.058 (9.21, 94.91) *	3.853 (1.84, 5.86) **	3.456 (0.42, 6.49) *
	NASH	95.872 (46.69, 145.06) ***	167.234 (89.32, 245.15) ***	82.528 (47.17, 117.89) ***	42.754 (−11.80, 97.31)	4.768 (2.48, 7.06)	3.938 (0.07, 7.80) *

SS: simple steatosis; bNASH: borderline non-alcoholic steatohepatitis; NASH: non-alcoholic steatohepatitis. Data are shown as the coefficient (95% CI). * *p* < 0.05; ** *p* < 0.01; *** *p* < 0.001.

**Table 2:** Linear regression models according to the fibrosis degree.

		IGFBP-1		IGFBP-2		IGFBP-7	
		Crude	Adjusted	Crude	Adjusted	Crude	Adjusted
Liver tissue	Exposure	−2.187 (−31.041, 26.668)	17.899 (−6.99, 42.79)	−11.963 (−23.80, −0.12) *	−9.707 (−23.96, 4.54)	1.768 (−6.34, 9.87)	4.398 (−4.95, 13.75)
	F0	43.925 (−30.11, 117.96)	108.685 (40.01, 177.36) **	167.126 (137.47, 196.78) ***	169.176 (130.50, 207.85) ***	2.012 (−18.63, 22.66)	8.565 (−17.24, 34.37)
	F1C	15.546 (−42.31, 73.40)	21.507 (−41.08, 84.10)	93.984 (69.98, 117.98) ***	81.854 (41.13, 122.58) ***	15.926 (−0.20, 32.06)	9.558 (−13.96, 33.07)
	F2	37.069 (−18.68, 92.82)	73.961 (9.41, 138.51) *	70.058 (46.91, 93.21) ***	52.506 (13.64, 91.37) **	28.996 (12.89, 45.10) **	29.142 (4.89, 53.39) *
Serum	Exposure	−14.280 (−39.42, 10.86)	−11.338 (−39.88, 17.21)	22.962 (6.02, 39.90) *	12.738 (−6.50, 31.977)	−0.230 (−1.24, 0.78)	−0.100 (−1.43, 1.23)
	F0	132.477 (67.16, 197.79) ***	173.399 (94.64, 252.16) ***	90.865 (46.85, 134.88) ***	46.646 (−6.435, 99.73)	1.722 (−0.90, 4.35)	2.057 (−1.60, 5.71)
	F1C	116.838 (67.17, 166.50) ***	169.778 (97.10, 241.56) ***	84.247 (50.78, 117.72) ***	49.147 (0.77, 97.52) *	4.740 (2.74, 6.74) ***	4.020 (0.69, 7.35) *
	F2	118.259 (69.09, 167.43) ***	184.027 (109.10, 258.06) ***	89.224 (56.09, 122.36) ***	61.460 (11.57, 111.35) *	4.566 (2.59, 6.54) ***	4.522 (1.08, 7.96) *

Data are shown as the coefficient (95% CI). * *p* < 0.05; ** *p* < 0.01; *** *p* < 0.001.

## Data Availability

Data are available upon direct request.

## Supplementary Materials

The following supporting information can be downloaded at https://www.mdpi.com/article/10.3390/medicina60030429/s1. Table S1: Linear regression models for IGFBP-3, -5, and -6 for NAFLD stage; Table S2: Linear regression models for IGFBP-3, -5, and -6 for fibrosis.
