# Supplementary material for: Insulin-like Growth Factor Binding Proteins and Cellular Senescence Are Involved in the Progression of Non-Alcoholic Fatty Liver Disease and Fibrosis in a Mouse Model
Source: Medicina (Kaunas). 2024 Mar 2;60(3):429. doi: 10.3390/medicina60030429 (PMC10972469; doi:10.3390/medicina60030429)
Supplement: Supplementary file 1 [file medicina-60-00429-s001.zip › medicina-2898334-supplementary.pdf]

Supplemental material

Supplemental table S1. Linear regression models for IGFBP-3, 5, and 6 for NAFLD stage

		**IGFBP-3**		**IGFBP-5**		**IGFBP-6**	
		Crude	Adjusted	Crude	Adjusted	Crude	Adjusted
Liver tissue	Exposure	-3.715 (-15.16, 7.73)	-0.815 (-13.831, 12.202)	76.984 (-63.239, 217.208)	136.507 (-27.673, 300.688)	-0.422 (-2.525, 1.681)	0.008 (-2.468, 2.484)
	SS	-23.496 (-53.157, 6.165)	-11.909 (-53.183, 29.365)	-386.005 (-749.416, -22.595)	-187.589 (-708.187, 333.009)	4.352 1.334, 10.037)*	6.655 (-1.455, 14.764)
	bNASH	15.938 (-6.126, 38.003)	41.29 (2.566, 80.015)*	-182.584 (-452.916, 87.748)	-282.957 (-771.391, 205.477)	-2.981 (-7.061, 1.099)	-6.176 (-12.78, 0.428)
	NASH	-22.531 (-47.735, 2.674)	-0.305 (-43.34, 42.73)	-356.795 (-665.605, -47.985)	-472.947 (-1015.752, 69.857)	-0.029 (-4.868, 4.81)*	-3.533 (-11.497, 4.431)
Serum	Exposure	-9.517 (-29.987, 10.952)	-0.101 (-21.689, 21.487)	-1.875 (-8.7, 4.951)	-4.819 (-13.316, 3.677)	6.172 (-2.785, 15.129)	5.418 (-2.948, 13.785)
	SS	4.093 (-49.118, 57.305)	66.479 (-8.477, 141.436)	-2.262 (-20.006, 15.482)	-5.376 (-34.876, 24.125)	32.021 (8.737, 55.306)	38.226 (9.177, 67.274)*
	bNASH	28.051 (-13.596, 69.698)	75.253 (21.3, 129.205)**	3.007 (-10.881, 16.894)	-8.289 (-29.523, 12.945)	20.915 (2.691, 39.139)	3.526 (-17.383, 24.435)
	NASH	42.14 (-5.264, 89.543)	103 (34.311, 171.689)**	-1.025 (-16.832, 14.782)	-11.586 (-38.619, 15.448)	9.488 (-11.255, 30.231)	-7.947 (-34.567, 18.673)

SS: Simple steatosis; bNASH: borderline non-alcoholic steatohepatitis; NASH: non-alcoholic steatohepatitits.

Data is shown as Coefficient (95% CI). *p<0.05; **p<0.01; ***p<0.001

Supplemental table S2. Linear regression models for IGFBP-3, 5, and 6 for fibrosis

		**IGFBP-3**		**IGFBP-5**		**IGFBP-6**	
		Crude	Adjusted	Crude	Adjusted	Crude	Adjusted
Liver tissue	Exposure	1.76 (-11.326, 14.847)	10.339 (-4.35, 25.029)	48.258 (-88.794, 185.309)	81.978 (-82.828, 246.785)	-1.22 (-3.691, 1.252)	-0.796 (-3.911, 2.32)
	F0	11.072 (-21.955, 44.098)	38.395 (-1.464, 78.254)	-451.667 (-797.543, -105.79)*	-347.092 (-794.291, 100.107)	0.318 (-5.979, 6.614)	0.643 (-7.954, 9.239)
	F1C	-19.236 (-45.968, 7.496)	-24.178 (-66.154, 17.798)	-422.031 (-701.985, -142.076)**	-442.473 (-913.421, 28.475)	-1.589 (-6.509, 3.331)	-3.587 (-11.421, 4.247)
	F2	-1.361 (-26.244, 23.523)	-1.856 (-41.915, 38.203)	-130.341 (-390.936, 130.254)	-70.778 (-520.213, 378.656)	-0.368 (-5.279, 4.543)	-1.576 (-9.655, 6.503)
Serum	Exposure	-6.794 (-29.925, 16.338)	-2.283 (-27.045, 22.48)	0.207 (-7.036, 7.451)	-0.136 (-8.803, 8.531)	2.626 (-7.432, 12.684)	3.855 (-6.841, 14.551)
	F0	24.852 (-35.245, 84.95)	63.512 (-4.81, 131.834)	12.847 5.972, 31.666)	7.361 (-16.552, 31.274)	10.92 (-15.212, 37.052)	8.859 (-20.651, 38.37)
	F1C	33.866 (-11.831, 79.564)	88.928 (26.663, 151.193)	0.08 (-14.23, 14.39)	-10.923 (-32.716, 10.87)	14.748 (-5.122, 34.619)	1.335 (-25.56, 28.229)
	F2	21.98 (-23.26, 67.219)	75.661 (11.446, 139.876)	-3.752 (-17.918, 10.415)	-21.603 (-44.078, 0.872)	28.017 (8.346, 47.689)**	19.274 (-8.462, 47.011)

Data is shown as Coefficient (95% CI). *p<0.05; **p<0.01; ***p<0.001.
